# Physiological responses to mask-associated CO_2_ exposure: a narrative review of acid–base balance, aging, and amyloidogenic stress

**DOI:** 10.3389/fpubh.2026.1759011

**Published:** 2026-03-02

**Authors:** Mawadda Alghrably, Farah Sukareh, Layla M. Khamis, Jordan Kahfi, Manel Dhahri, Abdul-Hamid Emwas, Mariusz Jaremko, Joanna Izabela Lachowicz

**Affiliations:** 1 Smart-Health Initiative (SHI) and Red Sea Research Center (RSRC), Division of Biological and Environmental Sciences and Engineering (BESE), King Abdullah University of Science and Technology (KAUST), Thuwal, Saudi Arabia; 2 Vaccine and Immunotherapy Unit, King Fahd Medical Research Centre, King Abdulaziz University, Jeddah, Saudi Arabia; 3 Biochemistry Department, Faculty of Science, King Abdulaziz University, Jeddah, Saudi Arabia; 4 Division of Biological and Environmental Sciences and Engineering, King Abdullah University of Science and Technology, Thuwal, Saudi Arabia; 5 Biology Department, Faculty of Science Yanbu, Taibah University, Yanbu El-Bahr, Saudi Arabia; 6 Department of Biology, College of Science, Taibah University, Yanbu, Saudi Arabia; 7 Core Labs, King Abdullah University of Science and Technology (KAUST), Thuwal, Saudi Arabia; 8 Department of Environmental Health, Occupational Medicine and Epidemiology, Wroclaw Medical University, Wroclaw, Poland

**Keywords:** acidosis, aging, amyloidogenesis, CO_2_ exposure, mask use, neurodegeneration, older adults, proteostasis

## Abstract

**Background:**

During the COVID-19 pandemic, prolonged mask use exposed billions of people to repeatedly elevated inhaled CO_2_ levels for extended periods. While these exposures typically produce only small pH shifts in healthy adults, older individuals exhibit age-related declines in respiratory, renal, metabolic, and proteostatic resilience that reduce their ability to buffer such disturbances. Because even mild acidosis can influence protein folding and accelerate amyloid formation under conditions of impaired homeostasis, aging populations may be disproportionately susceptible to downstream effects of chronic low-grade CO_2_ exposure.

**Methods:**

This narrative review synthesizes data on age-related changes in ventilation, acid–base regulation, metabolic buffering, and proteostasis, integrating these with biochemical pathways of pH-dependent amyloidogenesis. Evidence from mask-related CO_2_ exposure studies, protein-misfolding research, and gerontological physiology was analyzed to evaluate whether age-specific vulnerability could plausibly modulate amyloidogenic risk.

**Results:**

Across multiple studies, mask wearing increases inhaled CO_2_ concentrations and produces small but measurable reductions in blood pH in some conditions. Although these changes remain within normal physiological range in healthy adults, aging is associated with impaired ventilatory responsiveness to hypercapnia, diminished renal compensation, reduced muscle-based buffering due to sarcopenia, and mitochondrial and proteostatic decline. These changes lower physiological reserve and may magnify the biological impact of minor pH fluctuations. Experimental literature consistently demonstrates that acidity accelerates amyloid formation in proteins relevant to aging disorders—including Aβ, α-synuclein, IAPP, and β_2_-microglobulin—while older adults also accumulate comorbidities (chronic kidney disease, diabetes, neurodegeneration) that themselves predispose to acidosis and amyloidogenic stress.

**Conclusions:**

Although mask-associated CO_2_ elevations appear insufficient to induce amyloid formation in isolation, the combination of age-related physiological decline, chronic inflammation, impaired proteostasis, and reduced buffering capacity may heighten vulnerability in older adults. Given global demographic aging, further age-stratified research is needed to clarify long-term implications of repeated low-grade hypercapnia, refine diagnostic approaches for early detection of proteostatic stress, and develop prevention strategies tailored to aging physiology.

## 1 Introduction

The COVID-19 pandemic resulted in an unprecedented global adoption of face masks. In 2020, approximately 77% of countries implemented mandatory mask policies, and an estimated 4.5 billion people—nearly 58% of the world population—were required to wear masks in public, including children in schools (1). This scale and duration of population-wide mask use is without historical precedent and has prompted renewed scientific interest in potential physiological consequences, including prolonged exposure to elevated inhaled carbon dioxide (CO_2_).

Before the pandemic, several reports documented that certain mask types can increase inhaled CO_2_ concentrations, occasionally approaching or exceeding occupational exposure limits for acute (3% for 15 min) or chronic (0.5% for 8 h) inhalation (2–6). Human physiological adaptation to increased CO_2_ exposure involves acute ventilatory changes, with 1%−2% CO_2_ inhalation inducing an approximately 34% rise in minute ventilation (7). When CO_2_ accumulation surpasses compensatory capacity, respiratory acidosis may occur, triggering renal bicarbonate retention and increased H^+^ excretion to restore acid–base balance (8). Additional buffering adaptations—such as shifts in plasma electrolytes and bone-stored CO_2_–have also been described (9, 10).

**Figure 1:**
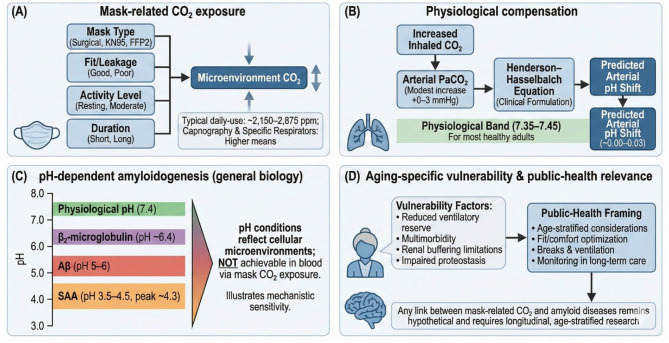
Conceptual framework linking mask-related CO_2_ exposure, physiological compensation, pH-dependent amyloidogenesis, and aging-specific public-health relevance. **(A)** Mask-related CO_2_ exposure. This panel illustrates the main determinants of CO_2_ accumulation in the mask microenvironment, including mask type (e.g., surgical, KN95, FFP2), mask fit and leakage, user activity level (resting to moderate exertion), and duration of wear. These factors influence the concentration of CO_2_ in the air immediately surrounding the nose and mouth. Reported typical daily-use microenvironment values range approximately from ~2,150 to 2,875 ppm, with higher levels observed during capnography and when using tightly fitted respirators. **(B)** Physiological compensation mechanisms. Inhalation of air with modestly increased CO_2_ results in a mild rise in arterial CO_2_ tension (PaCO_2_). The expected systemic acid–base response is illustrated using the Henderson–Hasselbalch equation, yielding a predicted arterial pH shift that generally remains within the physiologically normal range (7.35–7.45) for healthy adults. Estimated arterial pH changes shown are small (approximately −0.00 to −0.03), reflecting typical compensatory capacity. **(C)** pH-dependent amyloidogenesis (general biology). This panel summarizes the pH sensitivity of several amyloid-forming proteins (β_2_-microglobulin, Aβ peptides, and serum amyloid A), which exhibit enhanced aggregation kinetics under more acidic conditions in vitro. The gradient bar emphasizes that these pH thresholds reflect mechanistic findings from controlled systems and do not represent in vivo blood pH ranges expected from mask-related CO_2_ exposure. The panel is intended to contextualize biological sensitivity to pH changes rather than imply clinical risk. **(D)** Aging-specific vulnerability and public-health context. Older adults may exhibit factors that influence physiological resilience, including reduced ventilatory reserve, multimorbidity, impaired buffering capacity, and altered proteostasis. These considerations are relevant for public-health decision-making around long-term mask use, fit/comfort optimization, structured breaks, ventilation strategies, and monitoring in long-term care settings. The diagram notes that any link between mask-related CO_2_ exposure and amyloid diseases is speculative and requires rigorous longitudinal and age-stratified research to evaluate.

However, contemporary literature reveals conflicting findings regarding the physiological impact of mask-related CO_2_ accumulation. While some experimental studies report measurable increases in breathing-zone CO_2_, multiple systematic reviews—including an umbrella review published in 2025 (11) —have concluded that most mask types induce minimal to no clinically meaningful changes in respiratory rate, CO_2_ retention, or cardiopulmonary burden in healthy adults. Such discrepancies highlight the methodological heterogeneity across studies, including differences in mask design, activity level, sensor placement, exposure duration, and participant characteristics (Figure 1).

Importantly, the capacity to compensate for CO_2_-induced acid–base disturbances is not uniform across populations. Vulnerable groups—including older adults, children, pregnant women, and individuals with cardiopulmonary disease—may exhibit reduced ventilatory reserve and diminished physiological buffering (12, 13). This concern aligns with global public-health evidence showing that aging is associated with progressive loss of respiratory, renal, and metabolic resilience, as well as increased prevalence of multimorbidity and chronic inflammatory conditions that reduce homeostatic flexibility (Figure 1). Furthermore, contemporary gerontological reviews emphasize that aging populations experience disproportionate harm from environmental stressors, including heat, pollution, and air-quality fluctuations (14, 15). These insights are relevant when considering whether chronic, low-grade exposure to elevated CO_2_ during prolonged mask use could act as an additional physiological stressor in older individuals.

Chronic or intermittent CO_2_ exposure has been associated with cellular and molecular alterations, including inflammation, metabolic remodeling, disturbances in intracellular pH, and the formation of carboxylated protein derivatives (10) (Figure 1). Duarte et al. (16) hypothesized that persistent elevations in atmospheric or inhaled CO_2_ may dysregulate the proteome via pH-dependent mechanisms, potentially influencing pathways involved in protein folding, aggregation, and interaction dynamics.

Protein misfolding and aggregation are well-established contributors to numerous age-related diseases (17). More than 20 amyloidogenic proteins associated with disorders such as Alzheimer's disease, Parkinson's disease, type 2 diabetes, and AA amyloidosis have been identified (18–20). Environmental factors—including pH, temperature, metal ions, and ionic strength—can lead to destabilization of native protein conformations, promoting fibrillogenesis (21, 22).

Given that amyloid-related diseases are strongly age-dependent and that the global population is rapidly aging—with older adults increasingly concentrated in institutional environments where mask use may be prolonged—the intersection between chronic CO_2_ exposure, impaired proteostasis, and aging warrants careful examination. Recent public-health analyses confirm that older adults bear disproportionate morbidity from environmental exposures, including particulate pollution and climate-related stressors, underscoring the broader principle that age-related fragility magnifies the effects of otherwise modest physiological challenges.

This review highlights the complex interplay between CO_2_-related physiological changes, pH-dependent protein misfolding, and the age-related decline of proteostasis. While current evidence indicates that mask-associated CO_2_ exposure produces only small changes in arterial pH in healthy adults, aging markedly reduces ventilatory reserve, buffering capacity, mitochondrial resilience, and the functionality of chaperone-mediated and autophagic quality-control pathways. These age-related vulnerabilities may lower the threshold at which otherwise minor physiological stressors—including small pH fluctuations—become biologically meaningful. Given the accelerating demographic shift toward aging societies, understanding how chronic low-grade acidosis, impaired proteostasis, and environmental stressors interact to influence amyloidogenic risk is increasingly important for public-health planning.

This narrative review therefore explores the theoretical relationship between prolonged mask use, CO_2_-mediated pH alterations in biological fluids, and the molecular pathways of amyloidogenesis. We integrate (i) respiratory physiology under mask-related hypercapnia, (ii) pH-dependent mechanisms of protein misfolding, and (iii) population-specific vulnerabilities, with particular focus on older adults. The mechanistic connection between mild chronic hypercapnia and amyloid formation remains hypothetical; the aim of this review is not to claim causality but to evaluate whether available biochemical and epidemiological evidence justifies further investigation into chronic CO_2_ exposure as a potential modulator of proteostasis in aging populations.

## 2 Mask-wearing and pH decrease in biological fluids

Mask-induced airway resistance and CO_2_ rebreathing were experienced globally during the COVID-19 pandemic for more than 12 months, resulting in daily CO_2_ exposures that frequently exceeded short-term recommended limits. Consequently, the potential implications of long-term exceedance of safe exposure thresholds require careful evaluation. Fresh ambient air contains approximately 0.04% CO_2_, whereas wearing masks for more than 5 min has been shown to result in chronic exposure to inhaled CO_2_ concentrations ranging from approximately 1.41%−3.2% (13). When the mouth and nose are covered, the fraction of re-inhaled air increases, and CO_2_ concentrations may exceed 3%, while oxygen levels may decrease to around 17% (23), as summarized in a recent review detailing CO_2_ rebreathing associated with mask use (13).

There is accumulating evidence that mask wearing correlates with increases in blood CO_2_ levels, resulting in slight reductions in blood pH and mild acidosis. However, existing studies differ markedly in methodology, including mask type [surgical mask (SM), filtering face piece (FFP2), N95], duration of exposure (short-term vs. prolonged), sampling site (arterial vs. capillary blood), analytical protocols, and the physical activity performed during mask use (ranging from routine work in healthcare settings to controlled light or intense exercise across varying age groups and sexes). These methodological differences complicate direct comparisons across studies.

While some studies report measurable increases in CO_2_ microenvironment concentrations, convergent evidence from systematic reviews indicates that these elevations largely remain within compensatory capacity in healthy adults, highlighting the need for standardized measurement frameworks. To minimize bias, both supportive and non-supportive evidence must be considered, highlighting the need for standardized, long-duration CO_2_ monitoring protocols. The physiological significance of mask-related CO_2_ elevation therefore remains a subject of ongoing debate.

Grimm et al. (24) investigated 23 healthy volunteers and reported that both surgical mask (SM) and filtering facepiece class 2 (FFP2) masks significantly increased capillary blood pCO_2_ (SM: 35.2 ± 4.0 mmHg; FFP2: 34.5 ± 3.8 mmHg; *F* = 12.670, *p* < 0.001) and decreased capillary blood pH (SM: 7.39 ± 0.03 mmHg; FFP2: 7.39 ± 0.04 mmHg; *F* = 11.4, *p* < 0.001) during exercise compared to no-mask conditions (pCO_2_: 31.9 ± 3.3 mmHg; pH: 7.42 ± 0.03). The authors further noted that CO_2_ generation during intense short-term activity increased, while CO_2_ elimination was reduced when masks were worn, resulting in measurable disturbances of acid–base homeostasis and slight acidosis.

Long-term effects of mask use on blood gases were examined by Decha and Sonthaya (25) in 50 healthy adults performing running exercises while wearing reusable masks. Their findings demonstrated significant increases in arterial and red blood cell CO_2_ levels during mask use (*p* < 0.05). CO_2_ levels in the vigorous exercise group were significantly higher than in the moderate exercise group (*p* < 0.01). Despite these changes, arterial blood pH did not differ significantly before and after mask use (*p* > 0.05).

**Figure 2:**
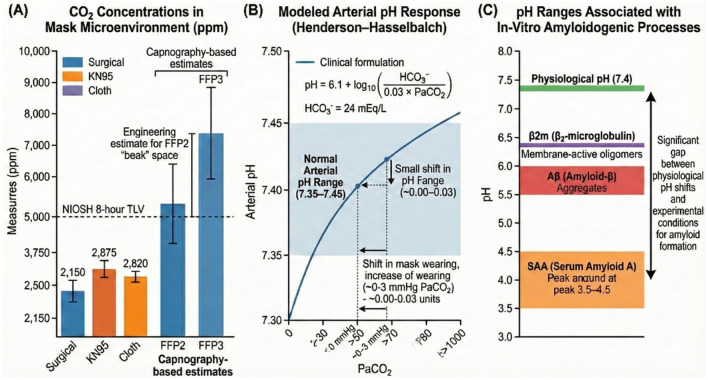
CO_2_ concentrations in mask microenvironments, modeled arterial pH responses, and pH ranges associated with in-vitro amyloidogenic processes. **(A)** CO_2_ concentrations in mask microenvironments. Measured and estimated CO_2_ levels are shown for surgical, KN95, cloth, FFP2 and FFP3 masks. Capnography-based measurements indicate typical microenvironmental CO_2_ concentrations between ~2,150–2,875 ppm for surgical, KN95, and cloth masks, while both capnography- and engineering-based estimates for FFP2 and FFP3 respirators indicate higher CO_2_ accumulation. The NIOSH 8-hour Threshold Limit Value (TLV) is marked for reference (132). Error bars represent standard deviations or reported upper/lower bounds from the source studies. No inferential statistical comparisons across mask types are included, as the displayed values originate from independent measurement datasets rather than a unified experimental cohort. **(B)** Modeled arterial pH response (Henderson–Hasselbalch equation). This panel depicts the predicted arterial pH response to incremental increases in arterial CO_2_ tension (PaCO_2_) using the standard clinical Henderson–Hasselbalch formulation, assuming ${\text{HCO}}_{3}^{-}$ ≈ 24 mEq/L. Small PaCO_2_ increases (approximately +0.3 to +3 mmHg) correspond to predicted arterial pH shifts of −0.00 to −0.03 units, remaining within the normal physiological range (7.35–7.45) (37). The curve represents a deterministic mathematical model rather than empirically tested data; therefore, no statistical tests, confidence intervals, or variance measures apply. **(C)** pH ranges associated with in-vitro amyloidogenic processes. Experimentally defined pH thresholds promoting aggregation of β_2_-microglobulin, amyloid-β, and serum amyloid A are shown alongside the physiological arterial pH (~7.4). This pH ranges represent published in-vitro experimental conditions, not in-vivo physiological environments. The displayed ranges reflect compiled values from multiple independent studies, not pooled data; therefore, no statistical comparison or variability metrics are applicable. The panel highlights the substantial separation between physiological arterial pH and the more acidic conditions required for in-vitro amyloid formation (86, 99, 109, 133, 134).

Several systematic reviews and controlled studies conclude that although mask use increases inhaled and exhaled CO_2_ concentrations, the resulting systemic CO_2_ levels in healthy individuals generally remain below National Institute for Occupational Safety and Health (NIOSH) limits for acute and chronic exposure, and do not induce clinically meaningful hypercapnia (4, 26) (Figure 2).

Recent work by Bao et al. (12), involving 30 healthy volunteers, evaluated the short-term physiological effects of wearing N95 masks during light and heavy exercise. The authors observed a reduction in venous pH, and calculated arterial pH demonstrated a downward trend following a 14-h masked intervention.

Conversely, Cebecioglu et al. (27) assessed respiratory function in 60 healthcare workers wearing surgical masks during the COVID-19 pandemic and reported no significant differences in venous blood pH, pO_2_, or pCO_2_ following at least 8 h of mask use. Venous blood samples collected before and after the work shift demonstrated stable values for pH, SpO_2_, PaO_2_, PaCO_2_, ${\text{HCO}}_{3}^{-}$, lactate, base excess, and oxygen saturation.

Ipek et al. (28) examined 34 healthcare workers who wore a surgical mask on the first day and an N95 mask on the following day for 1–4 h. Capillary blood gases obtained after mask removal revealed significantly higher pH values following N95 use (7.48 ± 0.04) compared to surgical mask use (7.43 ± 0.03) (*p* < 0.001). pCO_2_ levels were significantly lower after N95 use (28.46 ± 7.77 mmHg) than after surgical mask use (37.33 ± 8.81 mmHg) (*p* < 0.001). ${\text{HCO}}_{3}^{-}$ levels did not differ significantly between conditions (*p* = 0.113).

Tărăboanţă et al. (29) evaluated salivary parameters in 40 healthy participants after 2 h of wearing surgical and FFP2 masks. Measurements of unstimulated and stimulated salivary flow rate, pH, and buffer capacity taken on a non-mask day and after mask use revealed no statistically significant differences in salivary pH. To date, no data exists on long-term mask use and salivary pH.

The tear film protects the ocular surface by maintaining physiological pH (7.14–7.82), osmolarity, and electrolyte composition, with a secretion rate of approximately 2 μl/min ensuring continuous turnover (30). Exhaled air contains reduced oxygen and elevated CO_2_ levels, which can decrease tear film pH and potentially compromise ocular surface integrity (31). Corneal polymodal nociceptors—responsive to mechanical, thermal, and chemical stimuli—are activated by acidic solutions, with impulse frequency proportional to decreasing extracellular pH (32). Exposure of the ocular surface to CO_2_-rich air decreases corneal pH, thereby activating these nociceptors (33). During mask use, upward leakage of exhaled air with elevated CO_2_ content decreases tear film pH (30), impairs ocular surface homeostasis (31), and reduces stromal pH (34), collectively contributing to corneal pain sensations (35).

While ocular surface acidification provides a clear example of CO_2_ related pH reduction in a localized microenvironment, systemic acid–base regulation operates under different compensatory constraints, particularly in aging adults.

## 3 Tissue acidosis

**Figure 3:**
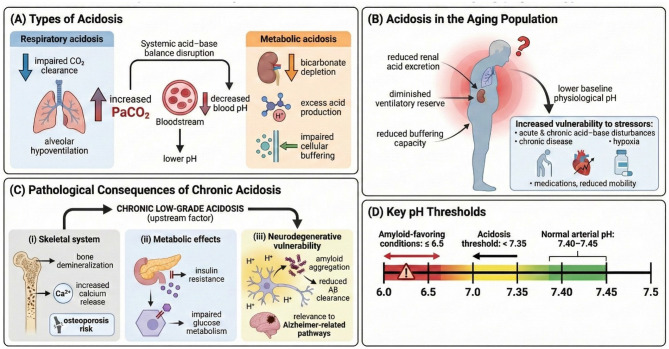
Conceptual summary of acidosis and its relevance to aging physiology. This figure synthesizes the major mechanisms by which acidosis develops, how aging increases susceptibility to acid–base disturbances, and the multisystem consequences of chronic low-grade acidosis. **(A)** Types of acidosis: Panel A illustrates the two principal categories of systemic acidosis. Respiratory acidosis arises from impaired CO_2_ clearance due to alveolar hypoventilation, resulting in increased arterial PaCO_2_ and a subsequent reduction in blood pH. In contrast, metabolic acidosis results from bicarbonate depletion, increased endogenous acid production, or impaired cellular buffering capacity. Both mechanisms converge on systemic acid–base imbalance and reduced extracellular pH. **(B)** Acidosis in the aging population: Panel B highlights age-associated physiological changes that heighten vulnerability to acid–base disturbances. Older adults exhibit reduced renal acid excretion, diminished ventilatory reserve, and an overall reduced buffering capacity. Together, these alterations lower the baseline physiological pH and blunt compensatory mechanisms. As a result, aging individuals demonstrate increased sensitivity to stressors—including acute and chronic illness, hypoxia, medication effects, and periods of reduced mobility—leading to more pronounced and prolonged acidosis compared with younger adults. **(C)** Pathological consequences of chronic low-grade acidosis: Panel C summarizes major organ-system effects of persistent, subclinical acidosis. (i) Skeletal system: Chronic acid load promotes bone demineralization through increased calcium mobilization, raising osteoporosis risk. (ii) Metabolic effects: Systemic acidosis impairs insulin signaling and glucose metabolism, contributing to metabolic inflexibility and insulin resistance. (iii) Neurodegenerative vulnerability: Acidic microenvironments favor amyloid aggregation and reduce amyloid-β clearance, potentially intersecting with Alzheimer-related pathways and accelerating neuronal vulnerability. **(D)** Key pH thresholds: Panel D depicts clinically relevant pH ranges. Normal arterial pH lies between 7.40–7.45, whereas values below 7.35 indicate systemic acidosis. The figure also shows that markedly acidic conditions (pH ≤ 6.5) produce biochemical environments that favor protein misfolding and amyloidogenic processes. This continuum emphasizes how modest shifts in pH can influence molecular stability and physiological resilience.

The body maintains acid–base homeostasis through coordinated regulation by the lungs, kidneys, and serum buffering systems. Tissue acidosis may arise from increased CO_2_ accumulation or from excess production or inadequate clearance of metabolic acids (36). Respiratory acidosis (pH < 7.35) develops (Figure 3) when CO_2_ production exceeds the capacity of the lungs to eliminate it (37). It is defined by elevated arterial CO_2_ tension (PaCO_2_ > 45 mmHg) resulting from alveolar hypoventilation (primary hypercapnia) (38). Respiratory acidosis may be acute, with bicarbonate (${\text{HCO}}_{3}^{-}$) increasing only 1 mEq/L for every 10 mmHg rise in PaCO_2_, or chronic (duration > 24 h), when renal compensation increases ${\text{HCO}}_{3}^{-}$ by approximately 4 mEq/L for every 10 mmHg rise in PaCO_2_ (39).

Metabolic acidosis (Figure 3) is typically a chronic condition characterized by retention of acid and depletion of bicarbonate stores, primarily affecting intracellular and interstitial compartments. Diagnosis requires assessment of fasting serum bicarbonate, urinary pH obtained ≥4 h after the last meal, and 24-h urinary citrate (40). Low-grade metabolic acidosis reduces systemic buffering capacity, increasing reliance on bone, muscle, and connective tissue to neutralize acid loads. This condition becomes more harmful with aging (Figure 3), as reduced renal function and diminished adaptive capacity impair the ability to maintain acid–base equilibrium. Even mild acidosis promotes urinary losses of sodium and potassium and accelerates bone demineralization by increasing osteoclast activity and reducing osteoblast function, thereby elevating the risk of osteoporosis and kidney stone formation.

Some individuals retain a normal systemic pH (7.35–7.45) despite deficiencies in buffering systems. Nevertheless, elevated intracellular and interstitial acidity may induce cellular injury, nociceptive signaling, and insulin resistance (41). Serum bicarbonate ≤24 mEq/L supports the diagnosis of low-grade metabolic acidosis, especially when associated with low urinary citrate or elevated urinary ammonium (42). Urinary pH values <5.3 in adults and <5.6 in children indicate metabolic acidosis (43).

In healthy tissues, extracellular pH averages ~7.4. However, interstitial acidification (pH < 6.5) is observed in inflammatory states, including solid tumors and infections (44). Intracellular pH is regulated through multiple buffering systems, such as Na^+^/H^+^ exchangers, Na^+^-driven ${\text{HCO}}_{3}^{-}$ transporters, Cl^−^/${\text{HCO}}_{3}^{-}$ exchangers, and ATP-dependent H^+^ pumps (45). Chronic extracellular acidosis disrupts cellular homeostasis, metabolism, signaling pathways, and gene transcription (46).

Acidosis (Figure 3) has been associated with ischemia, neuronal degeneration, chronic pain, and seizures (47). Acid-sensing ion channels (ASICs)—sodium- or calcium-permeable channels composed of three subunits (48)—are implicated in multiple neurological disorders, including Parkinson's disease, Huntington's disease, glioma, and seizure disorders (49). Hypoxia reduces pH and increases inflammatory mediators such as nitric oxide, arachidonic acid, and IL-1, all of which can activate or upregulate ASICs (49). Severe acidosis induces neuronal atrophy and membrane rupture, promoting rapid cell death. This leads to uncontrolled release of amyloid-β (Aβ), contributing to extracellular aggregation, plaque formation, and potential initiation of the Aβ cascade (50).

Acidosis may also contribute to the pathogenesis of vascular dementia and Alzheimer's disease (AD) (Figure 3). Low pH facilitates iron-catalyzed formation of reactive oxygen species by releasing iron from transferrin, ferritin, and other storage proteins (51). *In vivo* studies in 34 older adults demonstrated pH reductions in brain tissue using magnetic resonance spectroscopic imaging (52). Extracellular acidosis has also been detected in cerebrospinal fluid (53) and postmortem AD brain tissue (54). Blood acidification is a recognized feature of normal aging (55) and may reflect a broader age-related physiological process of declining acid–base resilience. Collectively, these findings suggest that brain acidification may represent a global phenomenon that worsens with advancing age, contributing to neurodegenerative vulnerability in older populations.

Acidosis may also intersect with diabetes-related pathways that accelerate AD onset and progression (Figure 3). Degradation of Aβ by insulin-degrading enzyme (IDE) is reduced at low pH (56). Additionally, both acute and chronic hypoglycemia cause neuronal injury through excessive glutamate and aspartate release, triggering calcium influx and decreasing intracellular pH (51).

Finally, excess human islet amyloid polypeptide (IAPP) represents a major pathological contributor to diabetic encephalopathy (DE). In patients with type 2 diabetes mellitus (T2DM), serum IAPP levels correlate positively with white-matter injury and negatively with cognitive performance. *In vitro* studies reveal that oligodendrocytes are more susceptible than neurons to acidosis induced by exogenous IAPP (57), underscoring a mechanistic link between metabolic dysregulation, acidification, and accelerated neurodegeneration in aging populations.

## 4 Aging, vulnerability, and CO_2_-related physiological stress

**Figure 4:**
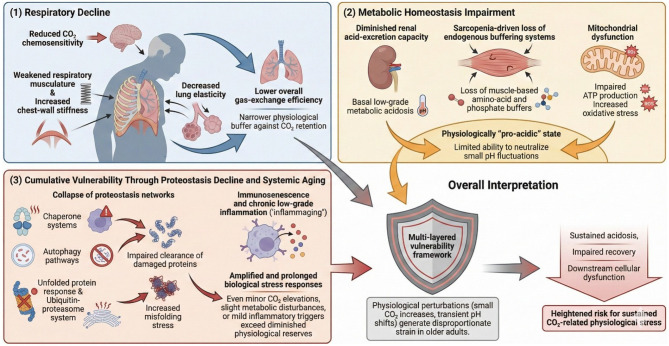
Aging-associated vulnerability and CO_2_-related physiological stress. This schematic illustrates the multidimensional mechanisms through which aging increases susceptibility to CO_2_-induced physiological disturbances. The framework integrates three major domains—respiratory decline, impaired metabolic homeostasis, and cumulative vulnerability due to proteostasis failure and systemic aging—and their convergence in promoting heightened biological strain in older adults. **(1)** Respiratory Decline. Age-related structural and functional deterioration of the respiratory system reduces the ability to effectively regulate CO_2_ levels. Key contributors include weakened respiratory musculature, increased chest-wall stiffness, and reduced elasticity of lung tissue. These changes collectively lower overall gas-exchange efficiency and blunt CO_2_ chemosensitivity. Diminished ventilatory responsiveness results in reduced physiological buffering against CO_2_ retention, thereby increasing vulnerability to transient or sustained hypercapnic challenges. **(2)** Metabolic Homeostasis Impairment. Aging disrupts acid–base balance through multiple metabolic pathways. Declines in renal acid-excretion capacity and reductions in amino acid- and phosphate-based endogenous buffering systems—partly driven by sarcopenia—contribute to a chronic shift toward a physiologically “pro-acidic” state. Mitochondrial dysfunction further exacerbates this imbalance by impairing ATP production and enhancing oxidative stress. Together, these processes limit the capacity to neutralize even small pH fluctuations, amplifying susceptibility to CO_2_-related physiological perturbations. **(3)** Cumulative Vulnerability through Proteostasis Decline and Systemic Aging. Global weakening of proteostasis networks—including reduced efficiency of autophagy pathways, chaperone systems, and the unfolded protein response—results in impaired clearance of damaged or misfolded proteins. Concurrent immunosenescence and chronic low-grade inflammation (“inflammaging”) heighten biological stress. These processes amplify and prolong cellular stress responses, reducing resilience to metabolic, oxidative, and acid–base disturbances. Consequently, even minor CO_2_ elevations may exceed physiological reserves in older adults.

Aging is accompanied by progressive physiological changes in the respiratory system (Figure 4) that diminish ventilatory reserve and reduce the capacity to maintain gas-exchange homeostasis under stress. These age-associated alterations affect multiple components of respiratory function, including chemosensitivity to CO_2_, the mechanical properties of the thoracic cage, alveolar–capillary diffusion efficiency, and renal acid–base compensatory mechanisms (Figure 4).

The age-related decline in respiratory, metabolic, and proteostatic resilience described here is reflected at the population level. Epidemiological analyses show that disability and chronic disease now account for the majority of years lived after age 60, and that age-structure effects alone explain nearly all projected increases in DALYs (58). Thus, the mechanistic vulnerabilities outlined in this work align with observed trajectories of functional decline in aging populations.

### 4.1 Aging and respiratory homeostasis

Recent studies demonstrate that aging markedly weakens ventilatory responsiveness to hypercapnia (Figure 4). A 2023 integrative review (59) of respiratory aging reports diminished respiratory muscle strength and attenuated ventilatory responses to both hypoxia and hypercapnia in older adults, reflecting a generalized decline in chemosensory function with age. Experimental work published in 2024 provides direct physiological evidence that cerebrovascular reactivity to CO_2_ is impaired in healthy older adults, with oxidative stress suppressing internal carotid artery dilation during hypercapnic exposure—an effect not observed in younger individuals (60). Collectively, these findings indicate that aging reduces both central and peripheral components of the CO_2_-driven ventilatory response, thereby decreasing respiratory adaptability during metabolic or environmental CO_2_ challenges.

Aging also leads to significant biomechanical restrictions of the thoracic cage. Multiple recent reviews document age-related reductions in lung elasticity, increased chest-wall stiffness, kyphotic spinal changes, and weakening of both primary and accessory respiratory muscles (59, 61–63). These structural alterations reduce chest-wall compliance and limit maximal inspiratory capacity. A 2023 review (61) highlights declining elastic recoil, reduced vital capacity, diminished respiratory muscle strength, and reduced ventilatory efficiency beginning as early as age 35–40. Contemporary geriatric respiratory physiology literature further confirms increased thoracic rigidity, reduced diaphragmatic strength, and decreased lung–chest wall compliance as primary determinants of lower ventilatory reserve in older adults (62).

The progressive loss of alveolar surface area and increased physiological dead space further compromise respiratory reserve (Figure 4). Age-associated reductions in diffusion capacity have been consistently reported. A 2023 review describes reduced gas-exchange efficiency and increased alveolar–arterial gradients due to parenchymal deterioration and senile-emphysema–like changes in older adults (**?**). Similarly, the Cambridge University Press geriatric critical care chapter reports decreased PaO_2_, increased A–a gradient, and reduced elastic recoil as hallmark gas-exchange impairments in aging, limiting the capacity to maintain adequate oxygenation during physiological stress (64).

In addition to respiratory constraints, aging impairs renal mechanisms of acid–base compensation (Figure 4). Renal adjustment to respiratory acidosis typically requires several days to reach full effect, as kidneys gradually modulate bicarbonate reabsorption and hydrogen excretion. Acid–base physiology resources confirm that while renal compensation begins within hours, it requires approximately 3–5 days to achieve maximal correction, a process further slowed by age-related decline in renal functional reserve (65). Contemporary analyses in respiratory physiology emphasize that aging reduces buffering capacity and acid-excretion efficiency, prolonging acidosis recovery during hypoventilation or CO_2_ retention (65).

Taken together, these findings show that aging diminishes respiratory reserve through linked impairments in CO_2_ chemosensitivity, thoracic compliance, gas-exchange efficiency, and renal buffering capacity. This combination reduces the ability of older adults to compensate for acute or chronic ventilatory challenges—including infection, reduced mobility, environmental stressors, or any condition that elevates CO_2_ production or retention. As the global population ages, recognizing these mechanistic vulnerabilities is critical for anticipating clinical risk, personalizing respiratory care, and improving public-health strategies.

### 4.2 Mitochondrial decline and metabolic acidosis

Beyond respiratory decline, aging is characterized by progressive alterations in metabolic homeostasis (Figure 4) that reduce the body's ability to maintain acid–base balance under stress. Evidence increasingly indicates that older adults frequently exhibit baseline low-grade metabolic acidosis, diminished buffering capacity due to sarcopenia, and impaired mitochondrial resilience—together creating a pro-acidic, energetically fragile physiological state.

With advancing age, renal function declines, reducing the capacity for metabolic acid excretion and bicarbonate regeneration. Recent studies describe how age-related reductions in renal acid excretion predispose older adults to chronic low-grade metabolic acidosis, particularly under Western dietary patterns characterized by high dietary acid load (DAL) (66). This acidosis often remains subclinical because serum pH may appear normal despite depleted bicarbonate stores. Clinical analyses emphasize that low-grade metabolic acidosis occurs intracellularly and within the interstitial space, even when blood pH remains within the reference range, with vulnerability increasing as renal reserve declines. Additional mechanistic studies suggest that persistent metabolic acidosis may accelerate aging through fibrosis promotion, klotho suppression, and altered telomerase activity—effects exacerbated in individuals with age-related kidney dysfunction (66).

Sarcopenia, a hallmark of physiological aging, is a major determinant of impaired acid–base buffering in later life. Muscle tissue represents the largest reservoir for amino acid- and phosphate-based buffers; thus, age-related muscle loss directly weakens systemic buffering capacity. Metabolic profiling of older adults reveals dysregulated fatty-acid oxidation intermediates and altered carnitine derivatives, indicating reduced metabolic flexibility and compromised buffering potential in sarcopenic muscle (67). Broader analyses describe widespread metabolic dysfunction in aging skeletal muscle, including altered amino-acid availability, impaired protein turnover, and increased inflammatory signaling, all of which impair endogenous acid neutralization (68). Gerontological reviews further describe sarcopenia as a metabolic syndrome of aging driven by hormonal decline, chronic low-grade inflammation, and redox imbalance—factors that diminish physiological resilience to acid accumulation (69).

Mitochondrial dysfunction (Figure 4) represents a central mechanism linking aging, metabolic acidosis, and sarcopenia. High-impact reviews note that aging skeletal muscle exhibits reduced mitochondrial biogenesis, elevated mitochondrial reactive oxygen species (mtROS), impaired mitophagy, and accumulation of mtDNA mutations, collectively diminishing energetic capacity under stress (70). Analyses from 2024 and 2025 further demonstrate that mitochondrial membrane instability, altered redox signaling, and defective quality-control pathways weaken metabolic resilience and heighten susceptibility to energy failure in older adults (71). Mechanistic studies show that mitochondrial dysfunction also impairs muscle stem-cell regenerative capacity, thereby sustaining sarcopenia progression and further compromising buffering function (72).

Together, these findings establish a reinforcing cycle in which renal decline, sarcopenia, and mitochondrial dysfunction collectively promote low-grade metabolic acidosis in older adults. Reduced renal clearance leads to acid retention; sarcopenia reduces buffering capacity; and mitochondrial dysfunction impairs ATP generation and redox balance necessary for compensatory metabolic responses. This metabolic vulnerability contributes to greater fragility, diminished stress tolerance, and heightened susceptibility to chronic diseases linked to acid–base imbalance.

### 4.3 Proteostasis collapse as a vulnerability amplifier

Proteostasis—the integrated system governing protein folding, trafficking, and degradation—also declines markedly with aging. This deterioration contributes to increased protein misfolding stress, impaired autophagic clearance, reduced chaperone function, and widespread weakening of the proteostasis network. Such deficits heighten vulnerability to metabolic dysfunction, neurodegeneration, sarcopenia, and systemic aging phenotypes (Figure 4).

Clinically, systemic inflammatory triggers such as SARS-CoV-2 infection amplify proteostasis network failure, increasing OD-like receptor pyrin domain-containing 3 (NLRP3) activation, oxidative burden, and tau- and Aβ-pathology progression, illustrating how stressors overwhelm aging proteostatic capacity (73).

Aging cells carry a substantially higher burden of misfolded and aggregated proteins. Mechanistic reviews identify proteostasis decay as a core hallmark of aging, characterized by impaired quality-control pathways and sustained stress within the protein-folding environment. Age-dependent decline in the ER unfolded-protein response (UPR) reduces the capacity to resolve misfolded proteins, leading to persistent ER stress and accumulating proteotoxic burden (74). Additional evidence shows that misfolded and aggregated proteins increase progressively with age due to declining chaperone activity, reduced UPS efficiency, and impaired autophagy pathways—each contributing to increased neurodegenerative risk (74).

Autophagy, the principal degradative mechanism for damaged proteins and organelles, undergoes consistent decline with age across multiple tissues. Human and mouse data reveal that chaperone-mediated autophagy (CMA) diminishes substantially with age, particularly in metabolically active tissues such as skeletal muscle. Loss of key CMA regulators, including lysosome-associated membrane protein type 2A (LAMP2A), leads to inefficient removal of misfolded proteins and contributes to myopathy, mitochondrial dysfunction, and loss of muscle contractility. A 2025 multi-organ analysis confirms widespread age-related CMA decline affecting stem-cell, neuronal, and metabolic compartments (**?**). Broader autophagy pathways (macro- and micro-autophagy) also exhibit age-related impairment, reducing the efficiency of eliminating damaged proteins and organelles; a 2023 review documents decreased functionality across all three autophagy subtypes with advancing age (75).

Molecular chaperones such as heat shock protein 70 (HSP70) and HSP90 play essential roles in preventing misfolding and aggregation. However, aging is associated with reduced chaperone levels and activity, impairing the capacity to maintain protein quality under stress (76). Decline of the multifunctional co-chaperone and ubiquitin-ligase CHIP further weakens misfolded-protein triage, reduces UPS activity, and promotes cellular senescence. These chaperone defects constitute one of the earliest drivers of proteostasis collapse (77).

The proteostasis network integrates the UPR, chaperone systems, the ubiquitin–proteasome system (UPS), and autophagy machinery. Aging disrupts this entire network. A 2025 review reports that ER proteostasis progressively fails with age due to weakened UPR sensors, impaired stress-repair mechanisms, and diminished adaptive capacity (78). Studies of CMA-deficient tissues show that age reduces compensatory proteolytic mechanisms, accelerating proteostasis failure, increasing oxidative stress, and reducing metabolic resilience compared with younger organisms (78). Another 2025 systems-biology analysis highlights proteostasis decline as a unifying mechanism across aging-related diseases, driven by UPS dysfunction, autophagy decline, loss of chaperone activity, and redox imbalance (79).

These proteostatic impairments amplify vulnerability to metabolic, inflammatory, and neurodegenerative damage. Elevated misfolding stress, loss of autophagic clearance, and impaired chaperone responsiveness contribute to the widespread functional decline characteristic of aging tissues. These failures interact with mitochondrial dysfunction, impaired stress-response pathways, and chronic inflammation, forming a self-reinforcing cycle that accelerates organismal aging.

Although CO_2_-induced pH shifts, protein-misfolding kinetics, and amyloidogenic processes are fundamental biological mechanisms across the lifespan, their severity, persistence, and downstream consequences are substantially magnified in aging organisms. Aging serves as a biological multiplier: small perturbations produce disproportionately large physiological effects due to diminished reserve. This heightened vulnerability reflects the progressive deterioration of buffering systems, proteostasis networks, immune resilience, and tissue-repair capacity.

Aging also reduces the ability to buffer physiological fluctuations in pH, CO_2_, and metabolic stress. Vascular physiology studies show that cerebrovascular responses to hypercapnia become slower, weaker, and less adaptable with age, indicating poorer ability to restore homeostasis after CO_2_ challenges that younger adults tolerate with little disturbance (80). These vascular changes interact with reductions in buffering capacity, mitochondrial resilience, and metabolic compensation, further enhancing the impact of even mild CO_2_-driven pH fluctuations.

Moreover, aging is characterized by immunosenescence and chronic low-grade inflammation (“inflammaging”), which jointly reduce the efficiency of damage resolution and increase tissue vulnerability. Aging immune systems exhibit diminished pathogen clearance, slower removal of damaged cells, and reduced stress resilience, making older individuals more susceptible to prolonged or amplified responses following mild external or physiological stressors (81, 82). These declines interact synergistically with proteostasis failure, accelerating proteotoxic and inflammatory cascades triggered by otherwise minor perturbations.

Collectively, these findings demonstrate that environmental or metabolic stressors easily tolerated by young, healthy adults can have amplified and persistent effects in older individuals. Aging is characterized by cumulative loss of buffering capacity, cellular repair efficiency, immune competence, and proteostasis robustness. As a result, even mild disturbances—small CO_2_ elevations, minor metabolic shifts, or low-grade inflammatory triggers—can exceed the reduced physiological reserve of older adults, producing heightened cellular stress, impaired recovery, and greater downstream biological impact.

## 5 Molecular mechanisms of amyloidosis and role of pH decrease

**Figure 5:**
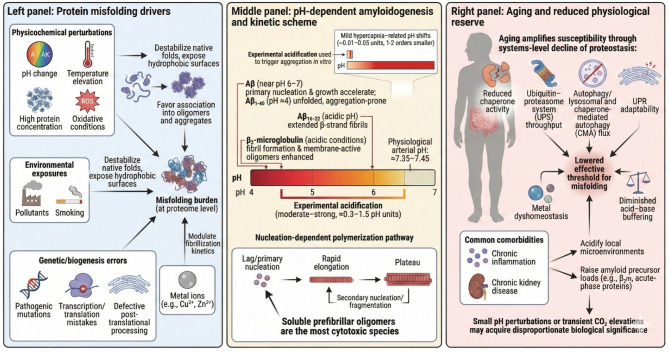
Mechanistic landscape linking physicochemical stressors, pH-dependent amyloidogenesis, and age-related proteostasis decline. The figure integrates three mechanistic domains illustrating how physicochemical perturbations, pH alterations, and aging-associated loss of proteostatic capacity converge to promote pathological protein aggregation. **Left panel:** Protein misfolding drivers. Multiple endogenous and exogenous stressors destabilize native protein conformations and favor amyloidogenic intermediates. Physicochemical perturbations—including pH shifts, temperature elevation, oxidative conditions, and increased protein concentration—expose hydrophobic residues and promote nucleation of misfolded structures. Environmental exposures such as pollutants and smoking similarly destabilize tertiary structures and enhance aggregation propensity. Genetic and biogenesis-related defects (e.g., pathogenic mutations, transcriptional or translational errors, and impaired post-translational processing) further compromise folding fidelity. Dysregulated metal ion homeostasis (e.g., Cu^2+^, Zn^2+^) modulates protein stability and aggregation kinetics, creating a permissive environment for nucleation. **Middle panel:** pH-dependent amyloidogenesis and kinetic scheme. Experimental acidification is shown as a central trigger of amyloidogenic conversion, with even minor reductions in pH (≤0.05–0.1 units) markedly increasing aggregation rates by several orders of magnitude. The panel compares the pH-dependent behavior of diverse proteins: amyloid-β (Aβ) shows accelerated primary nucleation and growth under mildly acidic conditions (pH 6–7), whereas Aβ_1−−40_ and Aβ_1−−42_ at pH ≤4 shift toward unfolded, aggregation-prone states. β_2_-microglobulin undergoes structural rearrangements in acidic environments that promote β-strand extension and fibril assembly; transthyretin (TTR) shows maximal aggregation near pH 4.0, coinciding with tetramer destabilization. The schematic summarizes the nucleation-dependent polymerization pathway, consisting of an initial lag/primary nucleation phase, a rapid elongation phase, and a plateau driven by secondary nucleation/fragmentation. Soluble prefibrillar oligomers are highlighted as the most cytotoxic species across amyloid systems. **Right panel:** Aging and reduced physiological reserve. Age-related decline in proteostasis reduces cellular and systemic resilience to stressors that would otherwise be buffered. Core proteostasis pathways—including the ubiquitin–proteasome system, molecular chaperone networks, autophagy–lysosomal degradation, and chaperone-mediated autophagy (CMA)—lose efficiency with age, while the unfolded protein response (UPR) exhibits diminished adaptability. This broad reduction lowers the effective threshold for protein misfolding, facilitates acid–base dysregulation, and promotes metal dyshomeostasis. Chronic conditions commonly associated with aging (e.g., chronic inflammation, chronic kidney disease, and diabetes) further exacerbate these deficits. The figure emphasizes that even small or transient pH perturbations, metabolic acidosis, or CO_2_ fluctuations can produce disproportionate biological consequences in older individuals, thereby amplifying susceptibility to amyloidogenesis.

Protein misfolding is driven by physicochemical stressors—including altered temperature, pH, protein concentration, and oxidative conditions—that destabilize native conformations and expose hydrophobic surfaces, promoting aggregation and precipitation (83) (Figure 5). Environmental factors such as pollutants and smoking can induce structural modifications, while genetic contributors include pathogenic mutations, errors in transcription or translation, impaired chaperone function, and defective post-translational processing. Aggregation propensity is additionally modulated by pH, protein concentration, and the presence of metal ions such as copper and zinc, which may accelerate or inhibit fibrillization depending on their concentration, coordination chemistry, and surrounding biochemical environment (83).

Experimental evidence shows that pH-dependent amyloidogenesis requires substantially larger acidification than occurs physiologically during mask-related CO_2_ exposure (Figure 5). Studies across multiple amyloid systems demonstrate that amyloidogenic proteins—including Aβ, IAPP, and β_2_-microglobulin—undergo conformational shifts, accelerated nucleation, or fibrillization primarily under moderate to strongly acidic conditions, requiring pH reductions of approximately 0.3–1.5 units. For example, Aβ primary nucleation accelerates as pH approaches its pKa near 7.0, with aggregation markedly faster at pH 6.0–7.0, while Aβ1-40 undergoes an unfolded, aggregation-prone conformation at pH ≈ 4. Similarly, Aβ16–22 peptides adopt extended β-strand fibril structures at acidic pH, promoting macroscopic assembly, and β_2_-microglobulin fibril–membrane disruption is strongly enhanced under acidic conditions, consistent with an acid-dependent shift in amyloidogenic behavior. By contrast, physiological evidence indicates that mask-associated CO_2_ retention produces only minimal arterial pH changes—typically 0.01–0.05 units in healthy individuals, because respiratory and renal homeostatic systems maintain blood pH tightly within 7.35–7.45 even during mild hypercapnia. These CO_2_-induced pH shifts are therefore one to two orders of magnitude smaller than experimentally induced acidifications used to trigger amyloidogenesis. Consequently, mask-related CO_2_ exposure alone is highly unlikely to reach amyloid-triggering thresholds, although individuals with modifying vulnerabilities—including chronic inflammation, which can acidify microenvironments and accelerate Aβ assembly, renal disease, which increases β_2_-microglobulin accumulation and promotes amyloidogenesis, or impaired proteostasis or genetic factors, which lower the threshold for misfolding stress—may exhibit a reduced margin of physiological protection (59–61, 64, 84, 85).

Amyloid fibril formation is a nucleation-dependent polymerization process in which hundreds to thousands of monomeric peptides assemble into elongated β-sheet-rich fibers (22). It proceeds through a slow primary nucleation lag phase, during which small, unstable oligomeric nuclei form, followed by rapid elongation, where monomers add to growing fibrils, and a final plateau once saturation is reached (Figure 5). Many proteins first convert into partially folded, aggregation-prone intermediates, allowing assembly without full unfolding; these intermediates can act as new nuclei that accelerate fibril growth (86). In an alternative secondary-nucleation model, fragmentation of existing fibrils generates additional growth points, markedly enhancing fibrillization (87). Both mechanisms are highly sensitive to environmental parameters, including pH, which can significantly accelerate aggregation. Importantly, growing evidence indicates that soluble prefibrillar oligomers, rather than mature fibrils, are the most cytotoxic species in amyloid diseases, as demonstrated for Aβ (88), tau fragments, huntingtin exon 1, and IAPP.

### 5.1 Chaperons and fibril formation

Protein function depends critically on maintaining a correct three-dimensional structure, yet many proteins struggle to reach or retain their native conformation when their folds are complex or intrinsically unstable (89). In such cases, molecular chaperones assist by stabilizing partially folded intermediates and guiding proteins back to their functional states—an observation that also reveals the natural tendency of some proteins to “flip” between low-energy functional conformations and non-functional or toxic states. Most misfolded proteins are ineffective or harmful, and cells therefore rely on several proteostasis mechanisms to prevent inappropriate folding and to eliminate misfolded species. Central to this system are chaperone proteins, which detect aberrant conformations, block β-sheet-driven aggregation, and direct terminally misfolded proteins toward cytoplasmic degradation pathways. Despite these safeguards, misfolding can still occur and underlies multiple protein-aggregation disorders. Notably, chaperone performance is itself sensitive to physicochemical conditions: the activity of α-crystallin declines significantly at low pH, whereas apolipoprotein J (clusterin) becomes more active under the same conditions (90).

The proteostasis network comprises an integrated system of molecular chaperones, ubiquitin-proteasomal degradation, lysosomal–autophagic pathways, and UPR signaling that collectively preserve protein folding integrity and prevent the accumulation of misfolded or aggregated species. Chaperones maintain nascent and stress-exposed proteins in a soluble, fold-competent state and triage damaged polypeptides for refolding or degradation, whereas autophagy–lysosomal systems remove larger aggregates and dysfunctional organelles that escape proteasomal clearance. The UPR, activated by endoplasmic-reticulum stress, adjusts protein-folding capacity and coordinates transcriptional programs essential for restoring proteome balance. However, UPR sensing and adaptive signaling decline with aging, reducing resilience to proteotoxic challenges. Together, these pathways form a dynamic surveillance system that buffers transient perturbations in protein stability and prevents misfolding-driven pathology. Under physiological conditions, several homeostatic mechanisms—including molecular chaperones, proteasomal degradation, autophagy-lysosomal pathways, and UPR signaling—prevent spontaneous aggregation despite transient pH shifts. Only when these systems are overwhelmed (aging, inflammation, genetic mutations) do small pH perturbations become more consequential (91–93).

### 5.2 Protein and peptide folding models

Protein misfolding often begins with pH-dependent destabilization of α-helical motifs, which can shift toward β-sheet–rich conformations that nucleate amyloid assembly (89). This helix-to-sheet transition is central to amyloidogenesis because β-sheet surfaces can recruit native proteins and template their conversion into pathogenic conformers, thereby amplifying misfolding within tissues. The resulting amyloid fibrils exhibit the characteristic cross-β architecture, in which β-strands align perpendicular to the fibril axis and form extended hydrogen-bonded sheets, ultimately assembling into protofilaments and mature fibrils. Although these structural motifs are universal, their formation is strongly influenced by the physicochemical environment, with pH being one of the most potent modulators of folding stability and aggregation propensity.

Decades of experimental work show that even modest acidification can expose aggregation-prone segments that are normally buried within native folds. Early studies demonstrated that poly-lysine, poly-glutamate, and oligo-alanine undergo secondary-structure transitions when pH shifts, while certain β-sheet peptides can revert to α-helices with pH or temperature changes. Several biologically important proteins undergo similar pH-dependent structural rearrangements. Influenza hemagglutinin, for example, undergoes a dramatic low-pH-triggered refolding, converting an unstructured region into an α-helix to mediate membrane fusion (94). The prion protein (PrP) is likewise highly sensitive to acidification: reduced pH increases mobility of the globular domain, disrupts the first helix, destabilizes the hydrophobic core, and accelerates conversion to the pathogenic PrP∧Sc-like conformation (95).

Islet amyloid polypeptide (IAPP) exhibits similar behavior. IAPP misfolding and fibril growth—central features in β-cell dysfunction and T2DM—are enhanced at lower pH, consistent with experiments showing pH-dependent exposure of amyloidogenic segments (96). Such pH-driven destabilization is further magnified when metal dyshomeostasis accompanies acidification. Lower pH can liberate transition metals such as Cu(II) and Zn(II) from binding partners, increasing concentrations of free ions that catalyze helix-to-sheet conversion in several amyloidogenic proteins, including PrP and IAPP (97). Although metal ions can either stabilize or destabilize protein structure depending on context, altered metal speciation at low pH is frequently associated with accelerated aggregation, particularly in neurodegenerative settings (98).

Clinical amyloidosis illustrates these principles. The aggregation of β2-microglobulin (β2m) in dialysis-related amyloidosis is strongly pH-dependent: fibril formation is markedly enhanced at low pH, requires partial unfolding, and is accelerated by fibril seeds (99). Likewise, membrane-active β2m oligomers that disrupt lipid bilayers form preferentially at acidic pH, whereas physiological pH favors dissociation into native, non-toxic monomers (100). Similar pH-sensitive behaviors are observed in lysozyme, which can form amyloids at physiological pH under pressure or at low pH and elevated temperature; in these systems, acidification shortens the lag phase and modulates fibril morphology (101). Aβ peptides also display striking pH-dependent aggregation kinetics: fibrils form within minutes at pH 5–6, whereas at pH 7.4 aggregation proceeds far more slowly and yields structurally distinct assemblies (134). Variants such as Aβ11-28 or Aβ11-23 further illustrate how pH interacts with metal-binding motifs (ATCUN) to modulate aggregation pathways.

Although these biochemical and biophysical phenomena apply broadly, their physiological impact is not uniform across the lifespan. Aging (Figure 4) is characterized by reduced proteostasis capacity, impaired metal homeostasis, declining chaperone activity, and diminished ability to maintain intracellular pH gradients. Consequently, older individuals experience greater structural destabilization in response to pH fluctuations, making them more susceptible to misfolding, oligomer formation, and downstream amyloid pathologies. Thus, while transient pH changes rarely induce misfolding in young, healthy systems, even mild acidification may carry greater pathogenic potential in aging tissues, especially under conditions of inflammation, oxidative stress, or impaired clearance.

## 6 Amyloidosis-related diseases and the role of pH in the protein aggregation process

Older adults have a higher prevalence of chronic conditions—chronic obstructive pulmonary disease (COPD), chronic kidney disease (CKD), diabetes, neurodegenerative disorders—that already compromise pH homeostasis and involve endogenous amyloidogenic proteins (Aβ, IAPP, β_2_-microglobulin). Even small additional acid-base stressors may therefore exacerbate ongoing misfolding pathways.

Amyloidosis presents with highly variable and often subtle symptoms, reflecting the specific organs in which amyloid accumulates. As deposits enlarge, they disrupt tissue architecture and function, producing a clinical spectrum ranging from mild, non-specific complaints to severe, life-threatening organ failure (102). Prognosis depends on amyloid type, organ involvement, and response to therapy; untreated systemic forms are typically fatal. Chronic inflammatory disease is a major driver of amyloid A (AA) amyloidosis, and delayed or missed diagnosis worsens outcomes. Cardiac infiltration markedly increases mortality, whereas gastrointestinal involvement—while less lethal—can cause nausea, diarrhea, or constipation.

pH-dependent aggregation of misfolded peptides and proteins is well documented in Alzheimer's disease, Parkinson's disease, and related neurodegenerative conditions (103). These observations underscore the need to better understand how changes in local acidity and immune activation may promote amyloidogenesis in other disorders, including ocular diseases such as cataract.

Contemporary classification of amyloidosis emphasizes the biochemical nature of the precursor protein. The most common systemic forms include amyloid light chain (AL) amyloidosis, caused by monoclonal light chains from clonal plasma-cell or B-cell disorders; AA amyloidosis, a complication of sustained elevation of serum amyloid A during chronic inflammation, leading to extracellular fibril deposition and progressive organ damage (104); and hereditary transthyretin amyloidosis (ATTR), an autosomal-dominant condition involving >130 transthyretin mutations, characterized by variable penetrance and severe cardiac and neurologic involvement (105).

These diverse forms of amyloidosis highlight how biochemical instability, inflammatory environments, and genetic predisposition interact to drive pathogenic fibril formation and organ dysfunction.

AL amyloidosis arises from misfolded immunoglobulin light chains that deposit in organs, causing progressive tissue damage and organ dysfunction. Kidney involvement occurs in roughly two-thirds of patients and frequently leads to renal failure, edema, and fatigue. Cardiac infiltration produces restrictive cardiomyopathy with dyspnea, arrhythmias, and high mortality. Recent *in vitro* work shows that AL fibrillogenesis begins with partial unfolding of the VL domain, dimer formation, and oligomerization through a multi-step β-sheet transition pathway (106). Aging increases susceptibility to AL disease by weakening proteostasis capacity and increasing systemic inflammation, both of which amplify light-chain misfolding risk.

AA amyloidosis results from chronic overproduction of serum amyloid A (SAA), typically driven by long-standing inflammatory disorders (107). SAA converts into extracellular amyloid fibrils that predominantly damage the kidney but may also involve the liver, spleen, thyroid, and bowel (108). SAA is intrinsically disordered; at acidic pH (3.5–4.5) it forms α-helical oligomers that convert into β-sheet structures on lipid membranes, suggesting a pH-dependent membranolytic mechanism (109). Aging—characterized by chronic low-grade inflammation (inflammaging) and impaired lysosomal clearance—further increases the likelihood of persistent SAA elevation and amyloid deposition.

ATTR amyloidosis is caused by >130 pathogenic TTR mutations leading to destabilized tetramers that misfold and aggregate extracellularly (105). Clinical manifestations include progressive peripheral neuropathy, autonomic dysfunction, and cardiomyopathy (110). Low-pH models show that acidic environments facilitate TTR tetramer dissociation and partial unfolding, accelerating fibrillization. Although stabilizing drugs like tafamidis improve TTR stability, they do not fully halt progression. Age further increases TTR amyloid risk due to declining proteostasis and reduced chaperone capacity, helping explain the strong association between ATTR and advanced age. LECT2 amyloidosis is now recognized as a major cause of renal amyloid deposition. LECT2 fibrillization requires loss of its bound Zn(II); zinc occupancy is strongly pH-dependent, dropping to ~20% at pH 6.5 (111). Aging-related changes in metal homeostasis and acidic microenvironments—common in chronic kidney disease—may therefore facilitate LECT2 dissociation and aggregation.

Mutations in the A-chain promote renal-dominant amyloid deposition, leading to proteinuria, hypertension, and azotemia, with occasional multiorgan involvement. Several TTR and A-chain mutants show markedly enhanced amyloid formation at moderately acidic pH, supporting a shared mechanism of pH-triggered destabilization and aggregation (112).

Cataract formation—responsible for most age-related blindness—results from aggregation of α-, β-, and γ-crystallins in the lens, a tissue that lacks protein turnover. Age-dependent covalent modifications, including deamidation, oxidation, and truncation, promote crystallin aggregation. pH-dependent destabilization also contributes: the βB1 R233H mutation, benign at neutral pH, significantly increases aggregation at acidic pH, implicating protonation-dependent destabilization (113). Such pH sensitivity aligns with the general decline of proteostasis and buffering capacity during aging.

Aging consistently amplifies susceptibility to systemic amyloidoses—including AL, AA, ATTR, ALECT2, hereditary forms—and to age-related protein-aggregation diseases such as cataract. Across these conditions, advancing age contributes to a progressive collapse of proteostasis, marked by declining chaperone activity, impaired proteasomal degradation, and reduced autophagy–lysosomal clearance, all of which heighten the cellular burden of misfolded or aggregation-prone proteins. Aging is also characterized by chronic low-grade inflammation, which elevates circulating SAA and immunoglobulin light chains, thereby increasing the substrate load for AA and AL amyloid formation. Concurrent decline in renal function reduces systemic buffering and accelerates acidotic microenvironments, conditions that promote amyloidogenic folding and worsen outcomes in AL and AA amyloidosis. Age-dependent metal dyshomeostasis, combined with localized pH shifts, further destabilizes protein structures and facilitates aggregation pathways implicated in neurodegeneration and crystallin aggregation in cataract. Compounding these effects, aging reduces the fidelity of protein-folding quality control, increasing proteome-wide insolubility and enhancing vulnerability to amyloid deposition across multiple organ systems. Collectively, these aging-related biological changes lower the threshold for amyloid formation and accelerate disease progression across diverse amyloid disorders.

## 7 Diagnosis of amyloid fibrils

Over the past two decades, major advances have transformed the early diagnosis of neurodegenerative disorders. Modern approaches now combine structural and functional neuroimaging, positron emission tomography (PET), biomarker-based assays, genetic sequencing, and digital physiological monitoring to improve diagnostic accuracy (114). Fluid-based biomarkers from cerebrospinal fluid, blood, serum, or saliva provide a less invasive and more cost-effective alternative to PET imaging for detecting molecular hallmarks of disease (115). In amyloidosis, the defining diagnostic signature is the cross-β fibrillar architecture, whose highly ordered, repetitive, and rigid structure allows detection through both *in vivo* and *in vitro* analytical techniques. Accurate diagnosis, classification, and staging require clinical assessment supported by electrocardiography and Doppler studies for cardiac involvement, manometry and gastric emptying tests for gastrointestinal dysfunction, and targeted blood or urine biomarkers. Ultimately, amyloid typing relies on tissue biopsy analyzed by immunohistochemistry or immunofluorescence, complemented by advanced structural methods such as solid-state NMR and cryo-TEM for fibril characterization.

### 7.1 *In vitro* detection of amyloid fibrils

Electron microscopy methods—including transmission electron microscopy (TEM), scanning electron microscopy (SEM), and atomic force microscopy (AFM)—consistently show amyloid fibrils as long, unbranched, and variably twisted fibers measuring approximately 7–20 nm in diameter. These fibrils share a characteristic X-ray diffraction signature, with a 4.7 Å meridional reflection corresponding to the spacing between β-strands and a 10 Å equatorial reflection reflecting the inter-sheet stacking distance. The cross-β architecture also underlies their strong binding to amyloid-specific dyes: thioflavin T (ThT), which yields enhanced fluorescence upon binding, and Congo red (CR), which produces a diagnostic absorption shift from ~490 nm to ~512 nm with an additional peak near 540 nm. Although atomic-level structural resolution was once limited to short peptide-derived fibrils, advances in solid-state NMR and cryo-TEM now allow detailed molecular characterization of full-length amyloid fibrils, providing deeper insight into their core architecture and structural polymorphism.

### 7.2 *In vivo* and clinical detection of amyloid fibril formation

Positron emission tomography (PET) tracers have been developed for early detection and classification of neurodegenerative disorders, targeting Aβ and Tau pathology in Alzheimer's disease and α-synuclein inclusions in Parkinson's disease; however, most tracers remain under clinical evaluation and are not yet widely implemented (116). Post-translational modifications (PTMs) of Tau, Aβ, and α-syn have been strongly linked to disease initiation and progression and are increasingly proposed as diagnostic and staging biomarkers (117). Comprehensive reviews highlight fluid-based assays—particularly Tau, Tau/Aβ42 ratios, and YKL-40 in serum—as promising, low-invasive markers for cognitive impairment (117). In amyloidosis, routine blood and urine tests are insufficient for diagnosis, although abnormalities such as hypoalbuminemia and hypercholesterolemia may appear in nephrotic syndrome (118). For suspected AL amyloidosis, bone marrow biopsy is essential to quantify plasma cells and exclude coexisting hematologic malignancies, while biomarkers such as NT-proBNP, troponin T, and free light chains provide prognostic information. Definitive *in vivo* diagnosis requires tissue biopsy from accessible sites such as subcutis, gastrointestinal mucosa, lips, or bone marrow (119). Congo red staining with polarized microscopy remains the standard method despite limited specificity, with alternative dyes—including FSB, alcian blue, luminescent oligothiophenes, and Thioflavin T (ThT)—offering complementary approaches. Following histological confirmation, amyloid typing is performed by immunohistochemistry or immunofluorescence, supplemented when necessary by mass spectrometry, immune electron microscopy, Western blotting, Enzyme-Linked Immunosorbent Assay (ELISA), or gene sequencing. Cardiac involvement is evaluated with electrocardiogram (ECG), where AL amyloidosis often presents with low voltage and prolonged *P*-wave conduction. Doppler and tissue Doppler imaging reliably detect diastolic dysfunction, and cardiac magnetic resonance imaging (MRI) has become a key diagnostic and prognostic modality. In ATTR amyloidosis, gastrointestinal symptoms are common, warranting evaluation with endoscopy, manometry, and gastric emptying studies (120).

## 8 Treatment and prevention of amyloidosis-related diseases

### 8.1 Therapeutic approaches

Effective treatment protocols for amyloidosis have become increasingly important as the incidence and global burden of these diseases continue to rise. Without advances in therapy, amyloidoses are projected to exert substantial clinical and economic strain due to progressive organ failure and associated mortality. Management requires a multidisciplinary approach, often involving hematologists, cardiologists, neurologists, nephrologists, gastroenterologists, and supportive-care specialists, depending on disease subtype and organ involvement.

Current therapeutic options remain limited, but major progress has been achieved in recent years. ATTR amyloidosis may be managed with liver transplantation to eliminate production of variant transthyretin (TTR). More recently, gene-silencing therapies have emerged as effective strategies for reducing hepatic TTR synthesis. Small-molecule TTR stabilizers—including tafamidis and diflunisal—along with siRNA and antisense oligonucleotide (ASO) therapeutics (patisiran, vutrisiran, inotersen) significantly decrease circulating TTR levels and slow disease progression (121). Clinical trials have demonstrated rapid, dose-dependent, and durable transthyretin reduction using ALN-TTR01 and ALN-TTR02 RNA-interference agents (ClinicalTrials.gov; NCT01148953, NCT01559077). Novel agents such as eplontersen (ASO) and CRISPR-Cas9 gene-editing approaches are currently under investigation (122, 123).

In AL amyloidosis, therapeutic success relies on rapid suppression of the amyloid-forming light chain. Standard regimens combine proteasome inhibitors, alkylating agents, immunotherapies, immunomodulators, and corticosteroids. Autologous stem cell transplantation (ASCT) with high-dose melphalan remains an option for selected patients. Treatment of AL cardiac amyloidosis requires optimal heart-failure therapy alongside clonal plasma-cell suppression (123), with cardiac transplantation reserved for carefully selected individuals. Emerging therapeutic approaches target both precursor reduction and amyloid removal. Agents such as epigallocatechin-3-gallate, doxycycline, and monoclonal antibodies (11-1F4, anti-SAP, NEOD001) have demonstrated potential to promote fibril resorption and improve organ outcomes (124).

For AA amyloidosis, control of the underlying chronic inflammatory condition remains paramount. Recent studies support the use of IL-1 suppression and SAA-silencing strategies (125), often combined with supportive organ-protective therapy to preserve quality of life.

In the field of neurodegeneration, research over the past two decades has accelerated the development of pharmacological therapies for Alzheimer's and Parkinson's disease (Supplementary Table S1). Growing attention has been directed toward the role of vitamin supplementation in neurodegenerative pathology. Vitamin D has been extensively reviewed in both Parkinson's (126) and Alzheimer's disease (127), with several clinical trials underway (ClinicalTrials.gov: NCT00571285, NCT01409694, NCT01420315). Vitamin E and other fat-soluble vitamins have been proposed as protective factors in PD (128) and AD (129), and a randomized trial showed that α-tocopherol reduced functional decline in mild-to-moderate AD. However, additional trials are ongoing (ClinicalTrials.gov; NCT0023571, NCT00040378, NCT01320527, NCT04491383), and evidence remains inconclusive for cataract prevention.

Overall, amyloidosis therapy is undergoing a paradigm shift from supportive care toward molecularly targeted, disease-modifying, and potentially curative strategies. Stabilizers, silencers, and gene-editing therapies are transforming ATTR management; updated chemotherapeutic regimens and monoclonal antibodies are reshaping AL treatment; and anti-inflammatory biologics remain central for preventing AA amyloidosis. Across all amyloid disorders, early detection, multimodal therapy, and combination approaches represent the most promising direction for reducing disease burden and improving long-term survival.

### 8.2 Prevention

Preventive approaches to reduce physiologic stress in vulnerable populations should emphasize optimization of respiratory ergonomics and environmental conditions. In older adults or individuals with impaired ventilatory reserve, actionable considerations include improving mask fit to minimize CO_2_ rebreathing, especially by reducing leaks around the nose and cheeks. Wider adoption of low–dead-space mask designs can further decrease CO_2_ accumulation and mitigate mild hypercapnic shifts. For individuals with reduced respiratory or metabolic buffering capacity, implementing periodic “mask breaks”—short intervals of unmasked breathing in well-ventilated or outdoor areas—may help prevent sustained CO_2_ retention. Preventive strategies should also incorporate monitoring of ambient ventilation and air exchange in classrooms, workplaces, and long-term care settings where masks are worn for extended periods. These measures collectively support safer mask use in physiologically vulnerable groups without compromising infection-control benefits.

Future pandemic preparedness will require more nuanced risk–benefit balancing when recommending prolonged mask use, particularly for aging populations with diminished respiratory adaptability. Public health guidelines should explicitly differentiate older adults from younger, healthier populations, acknowledging variation in respiratory mechanics, acid–base buffering, and proteostasis capacity. Age-stratified personal protective equipment (PPE) policies—especially in long-term care, rehabilitation centers, and geriatric outpatient facilities—may help reduce physiological strain while maintaining infection control. Policies should also include tailored recommendations for individuals with reduced ventilatory or metabolic buffering capacity, such as those with chronic kidney disease, heart failure, sarcopenia, or mitochondrial dysfunction. Incorporating these criteria into public health frameworks would support equitable guidance that balances infection-prevention benefits against physiologic vulnerabilities. As evidence accumulates, regulatory agencies may also consider incorporating design standards for low–dead-space masks and ventilation-compatible PPE specifically intended for high-risk groups.

### 8.3 Healthy aging and policy design: integrating molecular vulnerabilities with public health strategies

Healthy aging requires systems tailored to the needs of older adults, avoiding one-size-fits-all policies (130). As shown in populations with intellectual disability, aging interacts with long-standing biological and environmental vulnerabilities, leading to accelerated functional decline and greater susceptibility to health stressors (131). These findings parallel biological mechanisms outlined in this manuscript, where reduced proteostasis and metabolic buffering amplify the impact of small physiological perturbations.

Beyond the molecular and physiological mechanisms described in this manuscript, it is important to contextualize these findings within the broader public-health landscape of global aging. As highlighted by Gianfredi et al. (14), population aging is accelerating worldwide and is accompanied by rising burdens of multimorbidity, frailty, and functional decline, all of which increase physiological vulnerability to stressors. Healthy aging—defined by the WHO as the maintenance of intrinsic capacity across physical, cognitive, and social domains—declines as older adults accumulate chronic diseases, metabolic disturbances, and reduced resilience to environmental and biological challenges. These demographic trends reinforce the mechanistic observations presented in our work: weakened proteostasis, impaired mitochondrial function, decreased respiratory and renal compensation, and heightened inflammatory tone substantially narrow the physiological buffering range in older adults. Consequently, even modest metabolic or environmental perturbations—such as small pH fluctuations, transient CO_2_ retention, oxidative stress, or minor disruptions in metal homeostasis—may carry disproportionately greater biological consequences in aging populations. Integrating public-health evidence with molecular mechanisms thus underscores a central conclusion of this manuscript: aging simultaneously increases exposure to stressors and reduces the capacity to compensate for them, amplifying the risk that otherwise mild biochemical perturbations contribute to pathological misfolding, inflammation, and disease progression.

Demographic trends toward rapidly aging societies heighten the importance of considering how environmental stressors—including mild, chronic hypercapnia or low-grade acidosis—interact with age-related declines in organ resilience. As chronic diseases such as heart failure, chronic kidney disease, diabetes, and neurodegenerative disorders become increasingly prevalent, even small physiological perturbations may carry cumulative consequences. These interactions may compound existing burdens on healthcare systems over time, particularly if exposures exacerbate proteostasis decline, chronic inflammation, or mitochondrial insufficiency. Strengthening public health systems with early detection, longitudinal monitoring, and active surveillance for disorders influenced by impaired acid–base regulation or protein-folding instability will be essential. Tracking biomarkers of metabolic acidosis, renal function, and proteostatic stress could enable earlier intervention in at-risk individuals. Ultimately, integrating demographic forecasting with physiologic vulnerability profiles will be crucial to anticipate long-term population impacts and design prevention strategies that maintain health across the lifespan.

## 9 Conclusions

This narrative review synthesizes mechanistic, physiological, and public-health evidence to examine how chronic or intermittent exposure to mildly elevated CO_2_–such as may occur during prolonged mask use—interacts with aging-related declines in respiratory, metabolic, and proteostatic resilience. Current data indicate that mask-associated CO_2_ elevations in healthy adults generally remain within the range of physiological compensation and produce only minimal changes in arterial pH. These shifts are far smaller than those required experimentally to accelerate amyloid fibrillogenesis. Nonetheless, aging fundamentally reshapes the body's capacity to respond to metabolic and physicochemical stressors, narrowing homeostatic reserve across multiple systems. Declines in ventilatory responsiveness, renal buffering, mitochondrial efficiency, muscle-based metabolic support, and proteostasis collectively heighten vulnerability to even modest perturbations in pH, CO_2_, and oxidative or inflammatory load.

Across the lifespan, amyloidogenic proteins remain sensitive to acid–base conditions, metal dyshomeostasis, and proteostatic imbalance. While mild hypercapnia alone is unlikely to trigger amyloid formation, the cumulative interplay of chronic disease, inflammation, impaired clearance pathways, and aging-associated acidification may lower the threshold at which misfolding and aggregation become pathophysiologically relevant. This is particularly important given demographic trends toward rapidly aging populations, rising prevalence of chronic kidney disease, diabetes, and neurodegenerative disorders, and increasing numbers of older adults living in institutional environments where prolonged mask use may be routine.

From a public-health perspective, these findings underscore the need for age-stratified risk–benefit analyses when considering long-duration mask policies, personal protective equipment (PPE) design, and ventilation standards. They also highlight broader principles: aging increases exposure to physiological stressors while simultaneously diminishing resilience to them. As such, subtle environmental or metabolic challenges that are benign in younger adults may exert amplified biological effects in older individuals.

Clarifying the long-term implications of repeated low-grade hypercapnia, chronic acidosis, and impaired proteostasis requires rigorous, longitudinal, and mechanistically integrated research. Future studies should prioritize older adults and individuals with reduced ventilatory or renal reserve, incorporate standardized CO_2_ and pH monitoring protocols, and evaluate biomarkers of proteotoxic stress, inflammation, and metabolic compensation. Understanding how these factors intersect will be essential for guiding public-health strategies, optimizing protective equipment for high-risk groups, and ultimately safeguarding healthspan in aging societies.

## 10 Limitations

Several limitations must be acknowledged. First, available studies on mask-induced CO_2_ retention are heterogeneous in design, mask type, exposure duration, sampling methodology, and participant age, limiting direct comparability. Conflicting findings—ranging from measurable hypercapnic responses to no detectable systemic change—reflect inconsistencies in study protocols and measurement technologies. Second, most mechanistic insights into amyloidogenesis and pH-dependent protein misfolding derive from *in vitro* systems operating at far more acidic conditions than occur physiologically, making translational interpretation challenging. Third, data on older adults, individuals with chronic disease, and long-term mask users remain sparse. Finally, the speculative nature of linking chronic, mild hypercapnia to misfolding pathophysiology underscores the need for rigorous, longitudinal, and age-stratified research before drawing causal inferences.

## 11 Future directions

Future work should prioritize longitudinal and stratified clinical studies to evaluate CO_2_ retention, acid–base status, and proteostasis markers specifically in older adults, individuals with impaired renal or respiratory function, and those with chronic inflammatory or neurodegenerative conditions. Standardized protocols for measuring CO_2_ microenvironment levels, arterial and tissue pH, and biomarkers of proteotoxic stress are needed to reconcile existing discrepancies in the literature. Parallel experimental studies should investigate how mild, chronic acidosis affects aggregation kinetics of endogenous amyloidogenic proteins in physiologically relevant conditions and aging cellular models. Integration of multi-omics approaches—proteomics, metabolomics, and metallomics—may elucidate how aging-related changes in metal homeostasis, mitochondrial function, and protein-folding networks modulate susceptibility to misfolding.

At the population level, future research should evaluate risk-benefit frameworks for prolonged mask use in older adults and medically vulnerable groups. This includes studying the effects of mask design, fit optimization, ventilatory ergonomics, and structured break protocols on physiological outcomes. Public-health strategies may also benefit from the development of age-specific guidelines that incorporate physiological reserve, multimorbidity profiles, and proteostasis capacity.

## 12 Final perspective

Understanding how environmental stressors interact with aging physiology offers an important opportunity to refine preventive strategies for amyloidosis-related and other protein-misfolding disorders. As societies age, the identification of early biomarkers, individualized risk assessment, and tailored public-health recommendations will be essential for maintaining healthspan and mitigating long-term disease burden.
