# Supplementary material for: Physiological responses to mask-associated CO2 exposure: a narrative review of acid–base balance, aging, and amyloidogenic stress
Source: Front Public Health. 2026 Mar 2;14:1759011. doi: 10.3389/fpubh.2026.1759011 (PMC12990857; doi:10.3389/fpubh.2026.1759011)
Supplement: Supplementary file 1 [file Table_1.docx]

**Physiological Responses to Mask‑Associated CO₂ Exposure: A Narrative Review of Acid–Base Balance, Aging, and Amyloidogenic Stress**

Mawadda Alghrably^1^, Farah Sukareh^2^, Layla M. Khamis^3^, Jordan Kahfi^3^, Manel Dhahri^4^, Abdul-Hamid Emwas^5^, Mariusz Jaremko^1^, and Joanna I. Lachowicz^6,*^

*^1^Smart-Health Initiative (SHI) and Red Sea Research Center (RSRC), Division of Biological and Environmental Sciences and Engineering (BESE), King Abdullah University of Science and Technology (KAUST), Thuwal, 329555-6900, Saudi Arabia*

*^2^Vaccine and Immunotherapy Unit, King Fahd Medical Research Centre, King Abdulaziz University, Jeddah, Saudi Arabia; Biochemistry Department, Faculty of Science, King Abdulaziz University, Jeddah, Saudi Arabia*

*^3^Division of Biological and Environmental Sciences and Engineering, King Abdullah University of Science and Technology, Thuwal 23955-6900, Kingdom of Saudi Arabia*

*^4^Biology Department, Faculty of Science Yanbu, Taibah University, Yanbu El-Bahr 46423, Saudi Arabia*

*^5^Core Labs, King Abdullah University of Science and Technology (KAUST), Thuwal, 23955-6900, Saudi Arabia*

*^6^Department of Environmental Health, Occupational Medicine and Epidemiology, Wroclaw Medical University, Mikulicza-Radeckiego 7, 50-368 Wroclaw, Poland*

*Correspondance to: Joanna.lachowicz@umw.edu.pl*

**Table S1.** Overview of drugs currently used in treatment of Alzheimer disease, Parkinson disease, primary amyloidosis, and hereditary transthyretin amyloidosis (https://go.drugbank.com/).

| **Disease** | **Drug name** | **Drug description** |
| --- | --- | --- |
| Alzheimer | Aducanumab | A monoclonal antibody indicated in the treatment of Alzheimer's disease. |
|  | Cerebrolysin | A proteolytic protein indicated in the treatment of cerebrovascular disorders. |
|  | Donepezil | An acetylcholinesterase inhibitor used to treat the behavioural and cognitive effects of Alzheimer's Disease and other types of dementia. |
|  | Galantamine | A cholinesterase inhibitor used to manage mild to moderate dementia associated with Alzheimer's Disease. |
|  | Idebenone | An analogue of ubiquinone used for the treatment of visual impairment in adolescent and adult patients with Leber’s Hereditary Optic Neuropathy (LHON). |
|  | Lecanemab | An amyloid beta-targeting antibody used to treat Alzheimer’s Disease in patients with mild cognitive impairment or mild dementia with a known amyloid beta pathology. |
|  | Memantine | An N-methyl-D-aspartate antagonist (NMDA) receptor antagonist used to treat moderate to severe dementia in Alzheimer's. |
|  | Olanzapine | An antipsychotic drug used in the management of schizophrenia, bipolar 1 disorder, and agitation associated with these disorders. |
|  | Prazosin | An alpha-blocker that causes a decrease in total peripheral resistance and is used to treat hypertension. |
|  | Thioridazine | A phenothiazine antipsychotic used to treat schizophrenia and generalized anxiety disorder. |
|  | Trazodone | A serotonin uptake inhibitor used to treat major depressive disorder. |
| Parkinson | Amantadine | A medication used to treat dyskinesia in Parkinson's patients receiving levodopa, as well as extrapyramidal side effects of medications. |
|  | Apomorphine | A morphine derivative D2 dopamine agonist used to treat hypomobile "off" episodes of advanced Parkinson's disease. |
|  | Benserazide | A medication used to treat Parkinson's disease, parkinsonism, and restless leg syndrome. |
|  | Biperiden | A muscarinic receptor antagonist used to treat parkinsonism and control extrapyramidal side effects of neuroleptic drugs. |
|  | Bornaprine | An anticholinergic drug indicated in the treatment of hyperhidrosis, dyskinesia, akathisia, parkinsonism, and Parkinson's disease. |
|  | Bromocriptine | A dopamine D2 receptor agonist used for the treatment of galactorrhoea due to hyperprolactinemia and other prolactin-related conditions, as well as in early Parkinsonian Syndrome. |
|  | Carbidopa | A dopa decarboxylase inhibitor used in combination with levodopa for the symptomatic treatment of idiopathic Parkinson disease and other conditions associated with parkinsonian symptoms. |
|  | Droxidopa | A medication used to treat symptomatic neurogenic orthostatic hypotension (nOH) caused by dopamine beta-hydroxylase deficiency, non-diabetic autonomic neuropathy and primary autonomic failure caused by conditions such as Parkinson's disease. |
|  | Entacapone | A selective reversible catechol-O-methyltransferase inhibitor for the treatment of Parkinson's disease. |
|  | Istradefylline | A selective adenoside A2A receptor antagonist indicated in adjunct to levodopa and carbidopa for the treatment of Parkinson's Disease. |
|  | Levodopa | A dopamine precursor used in the management of Parkinson's disease, often in combination with carbidopa, as well as other conditions associated with parkinsonism. |
|  | Melevodopa | A methyl ester of levodopa indicated in combination with carbidopa for the treatment of Parkinson's disease. |
|  | Opicapone | A catechol-O-methyltransferase inhibitor used as an adjunct treatment for Parkinson's Disease in adults currently receiving levodopa and a dopa decarboxylase inhibitor. |
|  | Pergolide | A long-acting dopamine agonist that is uncommonly used for the management of Parkinson's disease, due to the risk for cardiac valvulopathy. |
|  | Piribedil | A dopamine agonist used with or without levodopa in the treatment of Parkinson's disease. |
|  | Pramipexole | A non-ergot dopamine agonist used to treat the signs and symptoms of idiopathic Parkinson's disease and Restless Legs Syndrome (RLS). |
|  | Quetiapine | A psychotropic agent used for the management of bipolar disorder, schizophrenia, and major depressive disorder. |
|  | Rasagiline | An irreversible inhibitor of monoamine oxidase used for the symptomatic management of idiopathic Parkinson's disease as initial monotherapy and as adjunct therapy to levodopa. |
|  | Ropinirole | A non-ergoline dopamine agonist used to treat the symptoms of Parkinson's disease and Restless Legs Syndrome. |
|  | Rotigotine | A non-selective dopamine agonist used for the treatment of Parkinson's Disease and Restless Leg Syndrome. |
|  | Safinamide | A MAO-B inhibitor used as an add-on treatment to levodopa/carbidopa for Parkinson's disease during "off" episodes. |
|  | Selegiline | A monoamine oxidase inhibitor used to treat major depressive disorder and Parkinson's. |
| Primary amyloidosis | Daratumumab | A CD38-directed cytolytic antibody used alone or as an adjunct drug in the treatment of multiple myeloma and light chain amyloidosis. |
|  | Lenalidomide | A thalidomide derivative used to treat multiple myeloma and anaemia in low to intermediate risk myelodysplastic syndrome. |
|  | Thalidomide | A medication used to treat cancers, particularly newly diagnosed multiple myeloma, and erythema nodosum leprosum. |
| Transthyretin amyloidosis | Tafamidis | A medication used to treat transthyretin-mediated amyloidosis. |
